# Whole-exome sequencing reveals candidate high-risk susceptibility genes for endometriosis

**DOI:** 10.1186/s40246-023-00538-9

**Published:** 2023-10-03

**Authors:** Susanna Nousiainen, Outi Kuismin, Siiri Reinikka, Roosa Manninen, Sara Khamaiseh, Mari Kuivalainen, Anna Terho, Sari Koivurova, Maarit Niinimäki, Kari Salokas, Markku Varjosalo, Anne Ahtikoski, Ralf Bützow, Outi Lindgren, Outi Uimari, Pia Vahteristo

**Affiliations:** 1 Applied Tumor Genomics Research Program, Research Programs Unit, University of Helsinki, Biomedicum Helsinki, Haartmaninkatu 8, P.O. Box 63, 00014 Helsinki, Finland; 2 Department of Medical and Clinical Genetics, University of Helsinki, Helsinki, Finland; 3 Department of Clinical Genetics, Oulu University Hospital, Oulu, Finland; 4 Research Unit of Clinical Medicine, University of Oulu, Oulu, Finland; 5 Medical Research Center Oulu, Oulu University Hospital, Oulu, Finland; 6 Department of Obstetrics and Gynecology, Oulu University Hospital, Oulu, Finland; 7 iCAN Digital Precision Cancer Medicine Flagship, Helsinki, Finland; 8 Department of Obstetrics and Gynecology, Kainuu Central Hospital, Kajaani, Finland; 9 Institute of Biotechnology, HiLIFE, University of Helsinki, Helsinki, Finland; 10 Department of Pathology, Turku University Hospital, Turku, Finland; 11 Department of Pathology, Helsinki University Hospital, Helsinki, Finland; 12 Department of Obstetrics and Gynecology, Helsinki University Hospital and University of Helsinki, Helsinki, Finland; 13 Department of Pathology, Oulu University Hospital, Oulu, Finland; 14 Research Unit of Population Health, Faculty of Medicine, University of Oulu, Oulu, Finland

**Keywords:** Endometriosis, Familial predisposition, Whole-exome sequencing, High-grade serous carcinoma, Candidate genes, FGFR4, NALCN, NAV2

## Abstract

**Background:**

Endometriosis is a common, chronic disease among fertile-aged women. Disease course may be highly invasive, requiring extensive surgery. The etiology of endometriosis remains elusive, though a high level of heritability is well established. Several low-penetrance predisposing loci have been identified, but high-risk susceptibility remains undetermined. Endometriosis is known to increase the risk of epithelial ovarian cancers, especially of endometrioid and clear cell types. Here, we have analyzed a Finnish family where four women have been diagnosed with surgically verified, severely symptomatic endometriosis and two of the patients also with high-grade serous carcinoma.

**Results:**

Whole-exome sequencing revealed three rare candidate predisposing variants segregating with endometriosis. The variants were c.1238C>T, p.(Pro413Leu) in *FGFR4*, c.5065C>T, p.(Arg1689Trp) in *NALCN*, and c.2086G>A, p.(Val696Met) in *NAV2*. The only variant predicted deleterious by in silico tools was the one in *FGFR4*. Further screening of the variants in 92 Finnish endometriosis and in 19 endometriosis–ovarian cancer patients did not reveal additional carriers. Histopathology, positive p53 immunostaining, and genetic analysis supported the high-grade serous subtype of the two tumors in the family.

**Conclusions:**

Here, we provide *FGFR4*, *NALCN*, and *NAV2* as novel high-risk candidate genes for familial endometriosis. Our results also support the association of endometriosis with high-grade serous carcinoma. Further studies are required to validate the findings and to reveal the exact pathogenesis mechanisms of endometriosis. Elucidating the genetic background of endometriosis defines the etiology of the disease and provides opportunities for expedited diagnostics and personalized treatments.

## Background

Endometriosis is a chronic, inflammatory disease where angiogenesis, fibrosis, invasion, and dissemination of endometrial-like tissue beyond the uterus are characteristic [1, 2]. Locations of endometriotic lesions include peritoneum and organs of the pelvic cavity, though ectopic endometrium can also grow elsewhere. The prevalence of endometriosis is estimated at 10%, resulting in approximately 190 million women with the disease worldwide [3]. Infertility and pain symptoms such as dysmenorrhea and dyspareunia are common and often require treatment: hormonal manipulation and, in some cases, surgical removal of the lesions [4]. Endometriotic lesions can be categorized as superficial peritoneal, ovarian cysts lined by endometrial epithelium (ovarian endometriosis), or deep lesions infiltrating into pelvic organs such as the bowel or bladder [5].

Based on twin studies, heritability of endometriosis is estimated at ~ 50% [6]. Several genome-wide association studies (GWAS) that aim to discover low-risk variants that are common in the population have been performed [7–11]. Multiple susceptibility loci have been identified, and for example, an association to *WNT4* locus has been replicated in many studies. The largest GWAS meta-analysis including 60,674 cases and 701,926 controls identified 42 significant loci for endometriosis predisposition, 31 of the loci being novel [12]. This meta-analysis highlighted the involvement of genes associated with pain perception or maintenance. Genetic correlations between endometriosis and 11 pain conditions and with inflammatory diseases were also observed. Overall, it has been estimated that common low-risk variants explain ~ 26% of the accountable variation [13].

In contrast with GWAS, only a few studies aiming at identifying high-risk predisposing variants have been conducted. These include two linkage studies, which highlighted chromosome regions 10q26 [14] and 7p13-15 [15] in endometriosis susceptibility. Targeted analyses on chromosome 7p13-15 identified an association to *NPSR1* [16]. Adding to evidence on high-risk susceptibility, familial cases of severe endometriosis have been reported: an Italian family of three sisters and their mother [17], two French families with multiple women suffering from deep endometriosis [18], and a Greek family of seven women in three generations with endometriosis and one of the patients also with adenomyosis [19].

Endometriosis has been associated with an increased risk for ovarian cancer. In a meta-analysis of 20 case–control and 15 cohort studies, a risk ratio (RR) of 1.27 [95% CI 1.21–1.32] was shown in the case–control studies and a standardized incidence ratio (SIR) of 1.80 [1.28–2.53] in the cohort studies [20]. Increased risk for endometrioid and clear cell carcinomas (RRs 1.76 [1.55–2.00] and 2.61 [2.23–3.05], respectively) and a decreased risk for serous carcinoma (RR 0.73 [0.62–0.87]) were seen in this meta-analysis. In a large cohort of 49,933 surgically verified Finnish endometriosis patients, the ovarian cancer risk (SIR) was 1.76 [95% CI 1.47–2.08] [21]. The highest risk was observed for endometrioid (3.12 [2.15–4.38]) and clear cell (5.17 [3.20–7.89]) histotypes, while the risk for serous histotype was 1.37 [1.02–1.80]. When the analysis was stratified by endometriosis type, ovarian endometriosis increased the risk for endometrioid and clear cell histotypes even more (4.72 [2.75–7.56] and 10.1 [5.50–16.9], respectively). Shared genetic risk factors with ovarian cancer were seen in genetic correlations (r_g_) between the diseases; significant correlations were observed for endometriosis and clear cell (0.71), endometrioid (0.48), and high-grade serous (0.19) histotypes [22].

Here, we report a Finnish family where four women in two generations have been diagnosed with surgically verified, hormonal treatment resistant endometriosis, and two of the patients also with high-grade serous carcinoma (HGSC). We utilized exome sequencing to identify potential causative gene defect(s) in this family.

## Study subjects and methods

### Study family

**Fig. 1:**
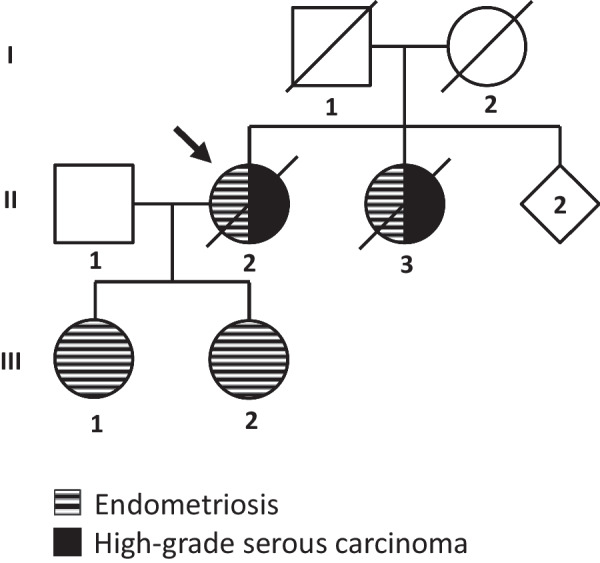
Pedigree of the Finnish endometriosis family. Four women in two generations have been diagnosed with surgically verified endometriosis and two also with high-grade serous carcinoma (HGSC). The index patient is marked with an arrow. The pedigree has been slightly modified for anonymity

In this study, we present a Finnish family where four close relatives have been diagnosed with endometriosis and two of the patients also with HGSC (Fig. 1). Before entering the study, the index patient underwent clinical genetic testing for inherited breast and ovarian cancer with negative results. The gene panel included, e.g., *BRCA1*, *BRCA2*, *TP53*, *CHEK2*, *PALB2*, *PIK3CA*, *PTEN*, and genes involved in mismatch repair.

A summary of gynecological diseases and hormonal treatments of individuals I-2, II-2, II-3, III-1, and III-2 is shown in Table 1. All four endometriosis patients have undergone multiple surgeries, as the course of the disease has been progressive despite hormonal treatment. Mother of the index (I-2) experienced heavy menstrual bleeding, and submucosal leiomyomas were suspected. She died at age 85. Index patient (II-2) had laparotomy at age 24 for ovarian endometriosis and was started on progestin-only pill. She had two pregnancies and vaginal deliveries under age 30. Hysterectomy was performed at age 35 due to heavy menstrual bleeding and leiomyomas. At age 50, she was diagnosed with HGSC (bilateral, ovaries) with omentum metastasis and extensive peritoneal carcinosis. Debulking surgery was performed followed by chemotherapy (docetaxel and carboplatin). Time to first recurrence was 2 years. She received chemotherapy again and relapsed for the second time 2 years later. She died at age 57, 7 years after the HGSC diagnosis. Sister of the index (II-3) underwent laparotomy for infertility investigation and had a left-sided salpingo-oophorectomy due to ovarian endometriosis at age 25. Ovarian endometriosis on the right side was resected, and adhesions and scarring in the pouch of Douglas were seen. At age 26, she had a suspected recurrent ovarian endometriosis, and at laparotomy, a serous cystadenoma on her right ovary was resected. A large pelvic mass on the right side was found at age 46 while investigating heavy menstrual bleeding and dysmenorrhea. Ovarian endometriosis was suspected, and she had her third laparotomy where her right-side adnexa were removed. The diagnosis was HGSC (tubal), and she underwent debulking surgery and chemotherapy (docetaxel and carboplatin). Time to recurrence was 18 months, followed by another cytoreductive surgery and chemotherapy. Soon after, she was diagnosed with metastasis and received chemotherapy and radiation therapy. She died at age 52, 6 years after the HGSC diagnosis. Daughter of the index (III-1) experienced primary dysmenorrhea and was treated with several progestin-only therapies. She had laparoscopy at age 24 and was diagnosed with peritoneal endometriosis, ovarian endometriosis cysts on her right ovary that were drained, and pelvic adhesions. After pregnancy and delivery, she had poor response to hormonal and pain treatment. On magnetic resonance imaging (MRI) at age 29, four ovarian endometriosis cysts on the right side, a deep lesion of 3 cm on cervix level, an intramural leiomyoma, adhesions, and apparent scarring distorting the positions of the uterus and vaginal fornix were seen. She opted for laparoscopic hysterectomy with salpingo-oophorectomy and salpingectomy and was continued with progestin-only pill postoperatively. The other daughter of the index (III-2) had her first laparoscopy at age 23, and peritoneal endometriosis was surgically treated, including salpingectomy. She then received hormonal treatments. At age 25, the pain symptoms had recurred. She had an MRI to evaluate the extensity of endometriosis and was diagnosed with deep (rectovaginal) endometriosis, ovarian endometriosis, and possible adenomyosis. She was started with gonadotropin-releasing hormone analog and later underwent laparoscopic resection of the sigmoid colon and pelvic adhesion liberation and was continued with other hormonal treatments.

**Table 1:** Summary of gynecological diseases and hormonal treatments in the study family

Individual	Endometriosis (age at dg)	HGSC (age at dg)	Uterine leiomyoma (age at dg)	Hormonal treatments
I-2	Not known	No	Suspected	Not known
II-2	Ovarian (24)	Stage IIIC (50)	Multiple (35)	Oral progestin
II-3	Ovarian (25)	Stage I (46)	No	No
III-1	Ovarian, peritoneal (24) Deep (29)	No	One (29)	Oral progestins, LNG-IUS, and GnRH analog injections
III-2	Peritoneal (23) Ovarian, deep (25) Suspected adenomyosis (25)	No	No	Oral progestins, GnRH analog injections, LNG-IUS, and combination oral contraceptive (continuous)

*HGSC* High-grade serous carcinoma, *LNG-IUS* Levonorgestrel-releasing intrauterine system, and *GnRH* Gonadotropin-releasing hormone

### Tissue samples

Blood-derived DNA samples from patients II-2, III-1, and III-2 were provided by the Department of Clinical Genetics, Oulu University Hospital (OUH, Oulu, Finland). Formalin-fixed paraffin-embedded (FFPE) tissue sample of HGSC from patient II-2, and HGSC and normal tissue (cervix) from patient II-3 were obtained from Biobank Borealis (BB, Oulu, Finland). For validation, DNA samples from 54 endometriosis patients and FFPE tissue samples from 19 patients with both endometriosis and a premalignant or malignant phenotype were obtained from BB. The biobank search terms included endometriosis and associated malignancies of serous, endometrioid, clear cell, and seromucinous carcinoma histologies in gynecological locations and their premalignant phases. In addition, we collected blood samples from 38 patients with deep endometriosis; these patients were treated at OUH or Kainuu Central Hospital (Kajaani, Finland), which are located in the same geographical region as the study family.

### DNA extraction

Archival HGSC sample from patient II-2 was macrodissected for DNA extraction resulting in tumor percentage of 40–50%. Tumor cell content of patient II-3 HGSC sample was 80–90%, and whole sections from the tissue block were cut for DNA extraction. DNA from FFPE samples was extracted with standard phenol–chloroform method utilizing MaXtract™ High-Density gel tubes (QIAGEN, Hilden, Germany). DNA was precipitated with NaCl and EtOH and dissolved in TE buffer.

### Whole-exome sequencing and sequence analysis

Target enrichment for whole-exome sequencing (WES) of the blood-derived DNA samples was performed using SeqCap EZ MedExome kit (Roche, Basel, Switzerland) and of the FFPE samples with KAPA HyperExome kit (Roche). Paired-end sequencing with 150-bp read length for blood-derived DNA samples and 75-bp read length for FFPE-derived DNA was performed with NextSeq 500 (Illumina Inc., San Diego, CA, USA) at Biomedicum Functional Genomics Unit (University of Helsinki). The quality of the FASTQ files was assessed with FastQC. Data were trimmed for low-quality bases at the ends of reads with Trimmomatic [23]. Genome Analysis Toolkit 4 (GATK4) [24] was used in the processing: The FASTQ files were aligned to the human reference genome GRCh38 with BWA-MEM, duplicate reads were removed with MarkDuplicates, and base quality scores were recalibrated with BaseRecalibrator and ApplyBQSR. Germline variants were called using HaplotypeCaller, and somatic variants of the carcinomas were called with Mutect2 against matched normal tissue samples. Non-synonymous and splice-site single-nucleotide variants (SNVs) and short insertions and deletions (InDels) of the WES data were analyzed and visualized using BasePlayer v1.0.2 [25]. Germline variants were filtered using gnomAD v3 and gnomAD v2 liftover for minor allele frequency (MAF) of 0.002 globally and in the Finnish population. An in-house panel of 40 normal (PON) WES samples was utilized to filter sequencing artifacts. The International Genome Sample Resource (IGSR) GRCh38 genome accessibility masks for 1000 Genomes data were used to filter out variants located areas of low mapping quality, such as segmental duplication areas [26]. We also filtered with a minimum coverage of four reads and a minimum allelic fraction of 25%. Somatic variants in the HGSC tumors were filtered with gnomAD v3, gnomAD v2 liftover, and an in-house PON with MAF 0. Minimum coverage of four reads and a minimum allelic fraction of 5% were required. SNVs located only at the ends of reads (defined as < 10 base distance) were discarded as artifacts. Exome data of the two HGSC samples were analyzed for somatic alterations in the most frequently mutated genes reported for this cancer type by The Cancer Genome Atlas (TCGA): *TP53*, *BRCA1*, *BRCA2*, *CSMD3*, *NF1*, *CDK12*, *FAT3*, *GABRA6*, and *RB1* [27]. The most commonly mutated gene in HGSC, *TP53,* was also manually evaluated for rare non-synonymous and splice-site variants from the BAM files of the HGSC samples utilizing BasePlayer. In addition, genes typically associated with ovarian endometrioid carcinoma (*CTNNB1*, *KRAS*, *ARID1A*, *PTEN*, *MLH1*, *MSH2*, *MSH6*, and *PMS2*) were analyzed.

### In silico prediction of the identified germline variants

The likelihood for a gene’s autosomal dominant inheritance was estimated with DOMINO (version Feb 19, 2019) [28]. Two in silico prediction methods were used to evaluate the pathogenicity of the observed variants: Combined Annotation-Dependent Depletion (CADD; GRCh38-v1.6) with a cutoff of > 15 [29] and Rare Exome Variant Ensemble Learner (REVEL; v1.3) with a cutoff of > 0.5 [30].

### Sanger sequencing

Candidate germline variants observed in WES were validated with Sanger sequencing. Primer sequences and PCR conditions are available upon request. Amplicons were sequenced at the Institute of Molecular Medicine Finland (FIMM) utilizing BigDye 3.1 chemistry. FinchTV v.1.4.0 (Geospiza Inc., Seattle, WA, USA) was used to visualize the chromatograms.

### p53 immunohistochemistry

p53 immunohistochemistry for the HGSC tissue samples was carried out at the Medical Research Center Oulu, Finland, using Dako (Agilent Technologies Inc., Santa Clara, CA, US) M7001 anti-human p53 antibody at 1:2400 dilution and BOND Polymer Refine Detection System DS9800 (Leica Biosystems, Deer Park, IL, US). The scoring was performed according to the WHO classification [31]: a strong and diffuse nuclear staining comprising > 80% of tumor cells, wild type, or total negativity in tumor cells. Both negative and excessive staining were considered pathogenic.

## Results

### Histopathology, p53 immunostaining, and genetic analysis support the HGSC diagnoses

**Fig. 2:**
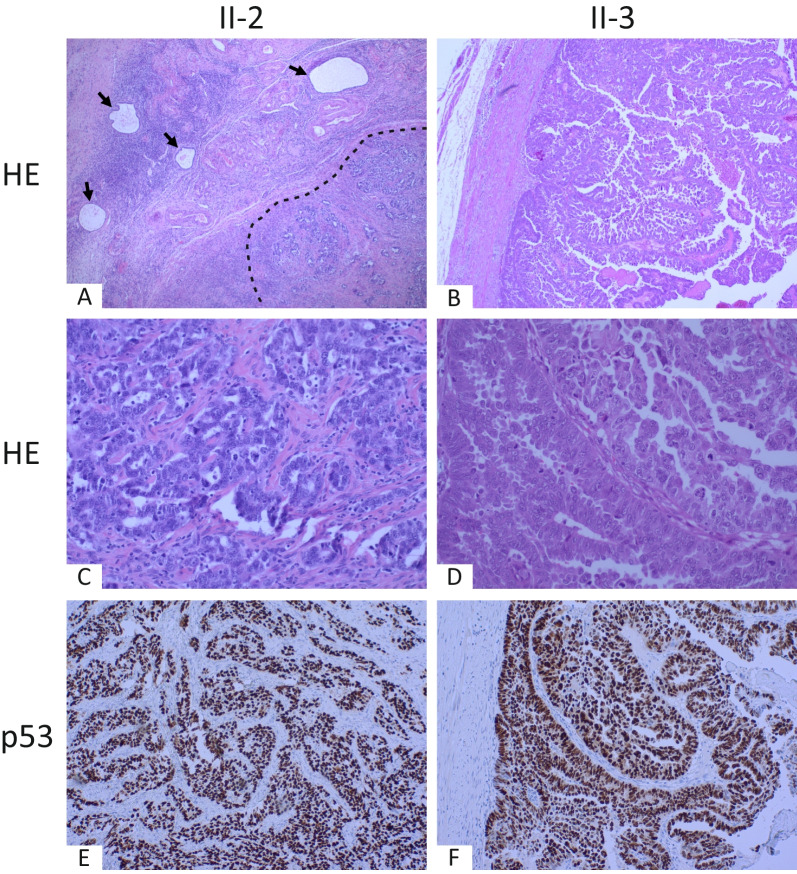
Histology and p53 status of high-grade serous carcinomas of individuals II-2 and II-3. **A** Dilated glands of endometriosis surrounded by endometrial type stroma (marked with arrows) and invasive glands of high-grade serous carcinoma (lower right quadrant, marked with a dashed line) in the ovary of individual II-2 (hematoxylin–eosin (HE) staining, original magnification × 4). **B** Serous carcinoma in the lumen of the fallopian tube of individual II-3 (HE staining, original magnification × 4). **C** Tumor epithelium with high-grade nuclear features and mitoses. Individual II-2 (HE staining, original magnification × 20). **D** Papillary structures of serous carcinoma with high-grade nuclear features and multiple mitoses. Individual II-3 (HE staining, original magnification × 20). **E**, **F** Strong p53 nuclear staining support the diagnosis of high-grade serous carcinoma of individuals II-2 and II-3, respectively (original magnification × 4)

Tumor histologies were re-evaluated from hematoxylin–eosin-stained tissue slides by an experienced pathologist (Fig. 2A–D). Both tumor samples displayed strong nuclear p53 immunostaining (Fig. 2E and F). In addition, no driver alterations in genes associated with endometrioid ovarian carcinoma (*CTNNB1*, *KRAS*, *ARID1A*, *PTEN*, *MLH1*, *MSH2*, *MSH6*, and *PMS2*) were identified in either tumor sample. All these results support the HGSC diagnoses of individuals II-2 and II-3.

### Exome sequencing reveals four germline variants that segregate with endometriosis

Sequencing coverage was determined using BasePlayer. The average coverage was 77 × for the blood-derived DNA samples and 110 × for the FFPE-derived DNA samples.

**Fig. 3:**
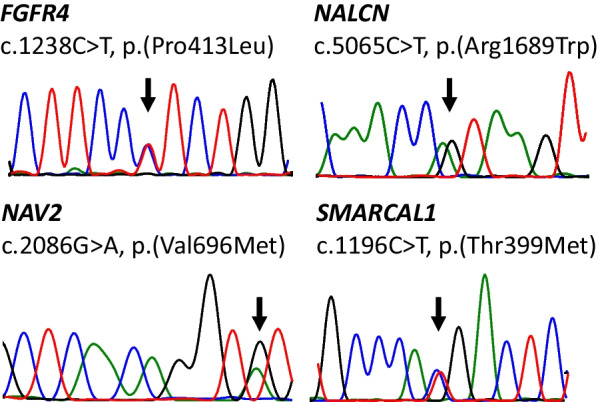
Sanger sequencing validates the segregating germline variants observed in exome sequencing. Images from the chromatograms of the blood sample of II-2 confirm heterozygous missense mutations in *FGFR4*, *NALCN*, *NAV2*, and *SMARCAL1*

Four heterozygous missense variants that segregate with endometriosis remained after filtering: c.1238C>T, p.(Pro413Leu) in fibroblast growth factor receptor 4 (*FGFR4*), c.5065C>T, p.(Arg1689Trp) in sodium leak channel, non-selective (*NALCN*), c.2086G>A, p.(Val696Met) in neuron navigator 2 (*NAV2*), and c.1196C>T, p.(Thr399Met) in SWI-/SNF-related, matrix-associated, actin-dependent regulator of chromatin, subfamily a like 1 (*SMARCAL1*) (Table 2). These germline variants were validated with Sanger sequencing (Fig. 3). The pathogenicity of the variants was evaluated with in silico tools CADD and REVEL. The only variant that was predicted likely pathogenic by both tools was the c.1238C>T, p.(Pro413Leu) in *FGFR4*. *SMARCAL1* variant c.1196C>T, p.(Thr399Met) received benign predictions with both tools and was associated with most likely recessive inheritance according to DOMINO; it was thus excluded from further analyses.

**Table 2:** Four germline variants that segregate with endometriosis were identified in exome sequencing

	*FGFR4*	*NALCN*	*NAV2*	*SMARCAL1*
*Variant information*				
Zygozity	Heterozygous	Heterozygous	Heterozygous	Heterozygous
HGVS coding^a^	c.1238C>T	c.5065C>T	c.2086G>A	c.1196C>T
HGVS protein^a^	p.(Pro413Leu)	p.(Arg1689Trp)	p.(Val696Met)	p.(Thr399Met)
Transcript^b^	ENST00000292408.9	ENST00000251127.11	ENST00000349880.9	ENST00000357276.9
*Population frequencies*				
Total^c^	0.00001598	0.0002192	0	0.001248
Finnish^c^	0.0001850	0.0007964	0	0.001075
*Predictions*				
Domino score^d^	0.586	0.364	0.303	0.089
CADD score^e^	27.7	26.6	13.84	0.041
REVEL score^f^	0.609	0.352	0.004	0.116

^a^Human Genome Variation Society

^b^Ensembl canonical transcript GRCh38.p13 release 108

^c^Allele frequencies in Genome Aggregation Database gnomAD v2.1.1 liftover

^d^Score is the probability of autosomal dominant inheritance P(AD)

^e^Combined Annotation-Dependent Depletion PHRED score. Values > 15 were considered likely pathogenic

^f^Rare Exome Variant Ensemble Learner. Values > 0.5 were considered likely pathogenic

Segregating variants in *FGFR4*, *NALCN,* and *NAV2* were screened in 92 individuals with endometriosis and in 19 individuals with both endometriosis and an associated tumor. Screening did not reveal additional carriers for any of the variants.

### Somatic cancer-associated mutations in the HGSC samples

The two HGSC samples were screened for somatic non-synonymous and splice-site mutations in genes previously identified relevant for HGSC by TCGA. The HGSC of patient II-2 harbored one low allelic fraction missense mutation in *TP53* (c.826G>C, p.(Ala276Pro)) (Table 3). The HGSC of patient II-3 displayed a high allelic fraction mutation in *TP53* (c.743G>A, p.(Arg248Gln)) and seven low allelic fraction mutations in four other TCGA HGSC genes. These included a splice-site mutation in *NF1 (*c.6819+1G>A) and six missense mutations in *CSMD3*, *CDK12*, and *FAT3*.

**Table 3:** Somatic mutations in HGSC genes^a^ identified in HGSC samples of patients with endometriosis and HGSC

	Gene	HGVS coding^b^	HGVS protein^b^	Canonical transcript^c^	AF^d^	COSMIC ID^e^
HGSC of II-2	*TP53*	c.826G>C	p.(Ala276Pro)	ENST00000269305.9	0.08	COSM43663
HGSC of II-3	*TP53*	c.743G>A	p.(Arg248Gln)	ENST00000269305.9	0.8	COSM10662
	*NF1*	c.6819+1G>A	NA^f^	ENST00000358273.9	0.11	COSM5096165
	*CSMD3*	c.9151G>T	p.(Asp3051Tyr)	ENST00000297405.10	0.06	NA^f^
	*CDK12*	c.3143G>T	p.(Arg1048Leu)	ENST00000447079.6	0.08	NA^f^
	*CDK12*	c.3375C>A	p.(Ser1125Arg)	ENST00000447079.6	0.08	NA^f^
	*FAT3*	c.1248T>G	p.(Asp416Glu)	ENST00000525166.6	0.06	NA^f^
	*FAT3*	c.10586C>T	p.(Pro3529Leu)	ENST00000525166.6	0.06	NA^f^
	*FAT3*	c.13321C>T	p.(His4441Tyr)	ENST00000525166.6	0.1	NA^f^

^a^HGSC genes: Relevant genes in high-grade serous carcinoma (HGSC) according to The Cancer Genome Atlas (TCGA)

^b^Human Genome Variation Society

^c^Ensembl canonical transcript GRCh38.p13 release 108

^d^Allelic fraction

^e^Catalogue of Somatic Mutations in Cancer, Legacy mutation ID v98

^f^Not applicable/not available

The two HGSCs were also screened for potential second-hit mutations in the four genes that harbor germline variants segregating with endometriosis in the family. One variant with a low allele frequency (AF 0.08, 3/39 reads) in *NAV2* (c.2018C>T, p.(Ala673Val)) was identified in the tumor sample of II-2.

## Discussion

Studying rare Mendelian disorders that share their phenotype with a common disease has been successful in elucidating the molecular background in these diseases; examples include familial forms of Alzheimer’s disease, diabetes, hypertension, and dyslipidemia [32]. It has been suggested that different changes in the same gene may lead to both rare and common forms of the disease. Finns, as a population, have gone through isolation and bottleneck events, resulting in genetic drift; combined with registries of genealogical and health data, this provides unique opportunities to identify rare monogenic diseases and their underlying genetic alterations [33]. Here, we have applied these approaches and analyzed a Finnish family with several individuals affected with endometriosis. These patients have been diagnosed at young age and shown debilitating symptoms. All patients have undergone multiple surgeries as hormonal treatments have failed to relieve the symptoms. We utilized hypothesis-free WES analysis and identified candidate high-risk variants in *FGFR4*, *NALCN,* and *NAV2* that segregate with the disease.

The only variant that was predicted pathogenic by both in silico tools was the alteration in *FGFR4*. Based on its’ known functions, *FGFR4* appears as a plausible candidate for endometriosis susceptibility. It is one of the four fibroblast growth factor receptors (FGFR1-4) that have been identified. When stimulated by fibroblast growth factors (FGF), these receptors drive cellular mechanisms involving proliferation, migration, and survival [34]. In the Catalogue Of Somatic Mutations In Cancer (COSMIC v98) [35], *FGFR4* is ranked as an oncogene in the Tier 1 Cancer Gene Census category of well-documented, relevant cancer genes [36]. Overexpression of FGFR4 and its ligand FGF19, as well as somatic mutations, have been identified in multiple cancers [37]. Also, a single-nucleotide polymorphism close to the variant identified in this study c.1162G>A (p.(Gly388Arg); rs351855) has been associated with tumorigenesis, progression, and prognosis [37]. In one study, FGFR4 overexpression was identified as a prognostic marker in advanced stage HGSC, and silencing of the receptor was shown to decrease cancer cell growth in vitro and in a mouse model [38]. In another study, FGFR4 expression was observed in ovarian cancer, especially in HGSCs of patients with residual disease after initial surgery [39]. The variant observed in this study has been reported in four individuals in the gnomAD database, all of whom are of Finnish descent. While in silico predictions and the known protein function provide *FGFR4* as a plausible candidate gene for endometriosis, additional studies are required to confirm whether this alteration is a real high-risk predisposing factor.

A rare segregating missense variant was identified in *NALCN*. This gene encodes for a cation channel that is part of a complex involved in regulating the resting membrane potential and excitation of neurons [40]. Specific germline mutations in *NALCN* are known to result in two neurological disorders; de novo heterozygous mutations to congenital contractures of the limbs and face, hypotonia, and developmental delay (CLIFAHDD [MIM 616266]), and biallelic mutations to infantile hypotonia with psychomotor retardation and characteristic facies 1 (IHPRF1 [MIM 615419]) documented in Online Mendelian Inheritance in Man (OMIM®) [41]. The p.(Arg1689Trp) variant observed in the endometriosis family is located far from the variants causing these inherited disorders. Recently, NALCN was identified as a regulatory protein in processes leading to cancer metastasis and in shedding of normal epithelial cells into circulation [42]. The authors found *NALCN* loss-of-function variant enrichment in human gastric and colorectal cancers and used murine models to demonstrate the deleterious effects with *NALCN* deletion or treatment with a NALCN channel blocker. In the gynecological setting, NALCN has been shown to participate in generating the leak current in human myometrial smooth muscle cells [43]. Knockout of smooth muscle-specific NALCN in mice reduced myometrial excitability and increased abnormal labor occurrence [44]. In addition, estrogen and progesterone response elements in the *NALCN* promoter have been identified, and these hormones have been shown to regulate NALCN expression and activity in human myometrial smooth muscle cells [45].

The *NAV2* variant identified in this study did not meet the pathogenicity thresholds in in silico predictions. However, the variant is very rare as it was absent in the gnomAD database. NAV2 belongs to a group of proteins called neuron navigators (NAV1-NAV3), homologues of well-studied *Caenorhabditis elegans* UNC-53, that are involved in actin cytoskeleton remodeling as in axon guidance [46]. Specifically, UNC-53/NAV2 has been linked to actin cytoskeleton dynamics via a linker protein ABI-1 to ARP2/3 complex [47]. The literature associating *NAV2* with gynecological functions or disease is scarce. Uterine leiomyosarcomas have been shown to overexpress *NAV2* when compared to endometrial stromal sarcomas [48], and it has been proposed as a candidate prognostic marker in uterine leiomyosarcoma [49].

Two endometriosis patients in the study family had been diagnosed with uterine leiomyoma (UL), and UL was suspected in one additional individual. Endometriosis and UL are both common, and their comorbidity has been shown in observational studies [50]. There is also genetic evidence linking these conditions. Four loci that have been reported in endometriosis susceptibility were identified also in a UL GWAS meta-analysis [51]. The shared loci (*WNT4/CDC42*, *GREB1*, *ESR1*, and *FSHB*) are involved in hormonal-signaling pathways. In addition, Mendelian randomization analyses suggest an overlap in the pathogenesis of these two diseases. Interestingly, a locus near *FGFR4* (rs2456181) was identified to associate with UL with heavy menstrual bleeding.

Two endometriosis patients in the study family had been diagnosed also with HGSC. There was a strong and diffuse nuclear p53 staining in the tumor cells of both tumor samples, confirming the diagnoses of HGSC according to the WHO classification. Exome sequencing of the HGSC of patient II-3 revealed a well-known *TP53* hotspot mutation (c.743G>A, p.(Arg248Gln); COSM10662). This mutation displayed a high allelic fraction, and it has been reported 1385 times in the COSMIC database, including multiple times in HGSC. All this indicates that the mutation has contributed to the observed aberrant p53 levels and HGSC formation. Also patient II-2 harbored a *TP53* mutation (c.826G>C, p.(Ala276Pro); COSM43663), but with the allelic fraction of only 0.08. Given that there is significant normal cell contamination in the sample (50–60%), the allelic fraction is likely higher in the tumor cell population. The variant has been reported in COSMIC 22 times, once in HGSC. While the tumor sample showed strong p53 expression, the effect of this mutation on protein level cannot be unambiguously concluded. For the rest of the TCGA HGSC genes, we identified low allelic fraction somatic mutations in *NF1*, *CSMD3*, *CDK12,* and *FAT3*. Their potential role on tumorigenesis remains obscure.

Over 90% of ovarian cancers are classified as epithelial ovarian cancers, with HGSC being the most common histological subtype [52]. Endometriosis-associated ovarian cancers (EAOC), namely, clear cell and endometrioid histotypes, are known to harbor recurrent somatic driver mutations, for example, in *ARID1A* and *PIK3CA* [53]*.* In contrast, somatic mutations in *TP53* are the main drivers in HGSC and can be found in 96% of the tumors [27]. Ovarian endometriosis and normal endometrium have been shown to harbor somatic mutations in EAOC-associated genes, with higher mutant allele frequencies in the endometriotic tissue, lineage tracing displaying clonal expansion [54]. Also, sequencing of deep endometriotic lesions has revealed known somatic cancer driver mutations in 26% of the samples, including mutations in *ARID1A* and *PIK3CA* [55]. In addition, rare, atypical cytological features harboring endometriotic lesions have been shown to increase the risk for synchronous or subsequent borderline or carcinoma tumors, with prevalence ranging from 12,2 to 25% in the cohorts [56, 57]. Despite cancer-associated mutations being relatively common in non-cancerous endometrial tissue, and malignant transformation is rare, accumulating evidence indicate endometriotic lesions as precursors for malignancy. The exact mechanisms for malignant transformation have not been unraveled yet, but may involve environmental and microenvironmental factors, somatic mutations, and germline predisposition. In our study family, two individuals had been diagnosed with endometriosis and HGSC. While this histotype has not been classified as a typical EAOC, our data indicate that HGSCs have evolved from endometriosis lesions. Whether the identified candidate genes for endometriosis predispose to HGSC per se cannot be concluded.

## Conclusions

Here, we have utilized exome sequencing and identified novel candidate genes for familial endometriosis. These include *FGFR4*, *NALCN*, and *NAV2*. Genetic validation in other endometriosis patients and/or populations is now required to confirm the findings. Our results also indicate that in addition to clear cell and endometrioid ovarian cancer, endometriosis is associated with the high-grade serous histotype. Identification of high-risk predisposing factors provides new insights in disease development, enables genetic testing, and offers novel treatment strategies urgently needed in endometriosis and ovarian cancer patient care.

## Data Availability

All main findings are presented in the manuscript. The raw data include sensitive germline data and are not publicly available due to confidentiality and compliance with the ethics statement.
